# The Dual Carrier ABSK System Based on a FIR Bandpass Filter

**DOI:** 10.3390/s140305644

**Published:** 2014-03-20

**Authors:** Zhimin Chen, Lenan Wu, Jiwu Wang

**Affiliations:** 1 Department of Information Science and Engineering, Southeast University, Nanjing 210096, China; E-Mail: wuln@seu.edu.cn; 2 Suzhou Easternwonder Information Technology Co., Ltd., Suzhou 215028, China; E-Mail: ewonder_jwwang@126.com

**Keywords:** impacting effect, slope-phase discrimination, dual carrier, ABSK signal

## Abstract

The special impacting filter (SIF) with IIR structure has been used to demodulate ABSK signals. The key points of SIF, including the resonance circuit's high Q value and the “slope-phase discrimination” character of the filter sideband, are demonstrated in the paper. The FIR narrow-band bandpass filtering system, which can also provide the impact-filtering effect, is proposed. A dual carrier system of ABSK signals is designed with the proposed FIR filter as its receiver. The simulation results show that the FIR filter can work well. Moreover, compared to the traditional SIF, the proposed FIR filter can not only achieve higher spectral efficiency, but also give better demodulation performance.

## 1. Introduction

With the rapid development of information technology, the traditional wireless communication systems which employ wasteful static spectrum allocation can no longer take full advantage of the spectrum. Meanwhile, the radio spectrum has become more and more complicated and fragmented. Therefore numerous efficient transmission techniques have been proposed to maximize utilization of the spectrum, which could also provide remarkable economic benefits [1–6].

The Asymmetry Binary Shift Keying (ABSK) modulation [7], proposed by Wu, is a typical kind of efficient transmission technique. There are two types of the modulation: the Extended Binary Phase Shift Keying (EBPSK) modulation and the EBPSK with Continuous Phase (CP-EBPSK) modulation. The modulation waveforms corresponding to code “0” and “1” have very tiny differences, which could be erased by a traditional finite impulse response (FIR) or infinite impulse response (IIR) filter. Therefore a special filter, called special impacting filter (SIF) [8], has been proposed for use as their receiver. The SIF with narrow bandwidth can produce high impact at the phase jumping point of the ABSK modulation waveform and greatly improve the output SNR, so threshold decision can be used next to the SIF to judge the code directly. Though the SIF receiver with IIR structure is easy to implement, the stabilization is not good. Because of the unstable performance of the SIF, we consider a FIR narrow-band bandpass filtering system instead.

## 2. The Mechanism of the SIF

Let *H*(*s*) be the system function, for the sake of notational simplicity, we denote:

(1) $$H(s)=\frac{K\overset{m}{\underset{j=1}{\text{∏}}}\left(s-z_{j}\right)}{\overset{n}{\underset{i=1}{\text{∏}}}\left(s-p_{i}\right)}$$

where K is a constant that doesn't affect the frequency response, Let *s* = *j*ω move along the imaginary axis, and we have:

(2) $$H(j\omega )=\frac{K\overset{m}{\underset{j=1}{\text{∏}}}\left(j\omega -z_{j}\right)}{\overset{n}{\underset{i=1}{\text{∏}}}\left(j\omega -p_{i}\right)}$$

**Figure 1.**
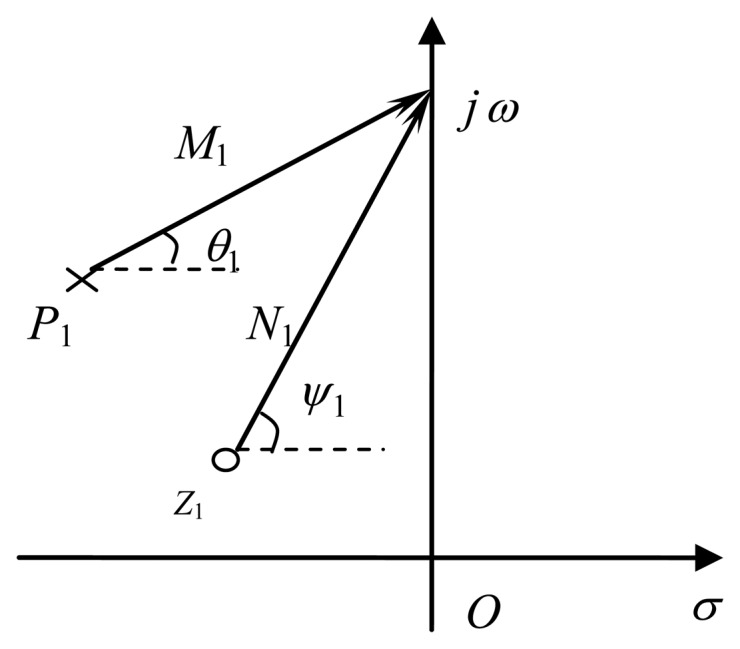
The vector of zeros and poles.

Note that the frequency response depends on the distribution of zeros and poles. The numerator and denominator mean the vectors from the zeros and poles to the point *j*ω on the imaginary axis, respectively, as shown in Figure 1.

*N*_1_ and *M*_1_ are the modules of the vector respectively, ψ_1_ and θ_1_ are the angles of the vector respectively. Without loss of generality, the complex factors of zeros and poles are given by:

(3) $$\begin{array}{ll} j\omega -z_{j} & =N_{j}e^{j\psi_{j}}\\ j\omega -p_{i} & =M_{i}{e^{j\theta }}_{i} \end{array}$$

Equation (2) can be rewritten as:

(4) $$\begin{array}{ll} H(j\omega ) & =K\frac{N_{1}e^{j\psi_{1}}N_{2}e^{j\psi_{2}}\cdots N_{m}e^{j\psi_{m}}}{M_{1}e^{j\theta_{1}}M_{2}e^{j\theta_{2}}\cdots M_{n}e^{j\theta_{n}}}\\ & =K\frac{N_{1}N_{2}\cdots N_{m}}{M_{1}M_{2}\cdots M_{n}}e^{j[(\psi_{1}+\psi_{2}+\cdots +\psi_{m})-(\theta_{1}+\theta_{2}+\cdots +\theta_{n})]}\\ & =\left|H(j\omega )\right|e^{j\phi (\omega )} \end{array}$$

where:

$\left|H(j\omega )\right|=K\frac{N_{1}N_{2}\cdots N_{m}}{M_{1}M_{2}\cdots M_{n}}$ is the system function's module;
*φ*(*ω*) = *e^j^*^[(ψ^_1_^+ ψ^_2_^+…+ ψ^*_m_*^)−(θ^_1_^+θ^_2_^+…+θ^*_n_*^)]^ is the phase.

With ω moving along the imaginary axis, the module and angle change as well, the relationship between module and phase could be explained by the following explanation. If the circuit has high Q value and the transfer function has a couple of poles and zeros adjacent to the imaginary axis respectively, that is:

(5) $$\begin{array}{ll} \text{Zeros}:z_{j}=-\sigma_{j}\pm j\omega_{j}, & \left(\sigma_{j}\ll \omega_{j}\right) \end{array}$$

(6) $$\begin{array}{ll} \text{Poles}:p_{i}=-\sigma_{i}\pm j\omega_{i}, & \left(\sigma_{i}\ll \omega_{i}\right) \end{array}$$

**Figure 2.**
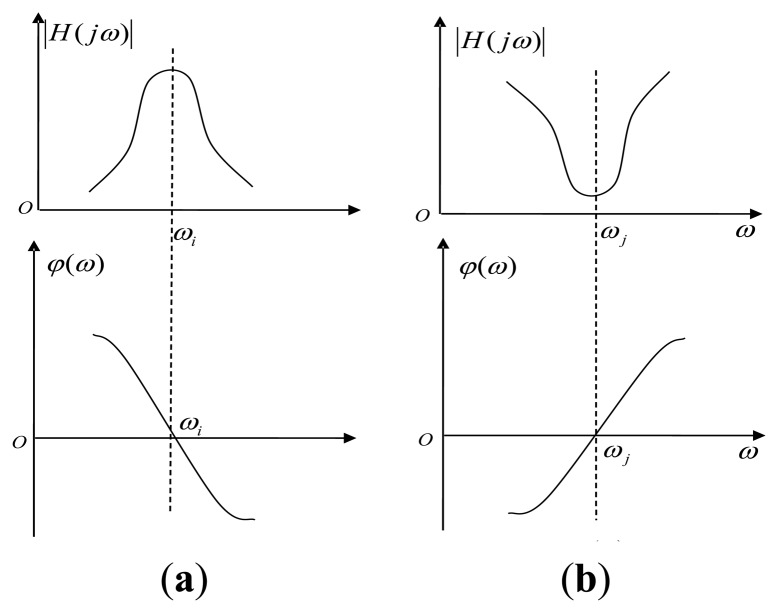
Frequency response of zeros and poles adjacent to *j*ω axis.

The amplitude-frequency response reach a peak and the phase-frequency response decrease rapidly when the poles approaching the point ω = ω*_i_*; when the zeros approach the point ω = ω*_j_*, the result is just the opposite, as shown in Figure 2a,b, respectively.

So we conclude that the steeper transitional band of the amplitude-frequency curves, the steeper of the phase-frequency curves [9]. If the frequency *f_d_* is located in the biggest slope of the phase-frequency curve, the tiny change of phase will bring big change of the frequency. The big range frequency shift mapping in the amplitude-frequency curves would show the amplitude changes fiercely, we call it “slope-phase discrimination”.

**Figure 3.**
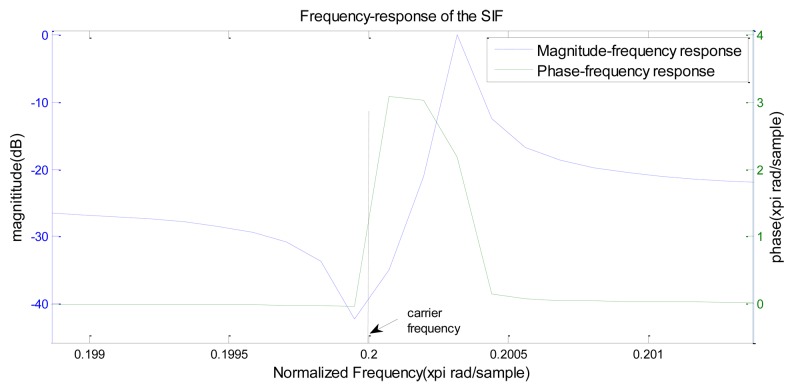
Magnification of one segment of the SIF's frequency response.

At present the SIR which has one or more conjugate zeros and multi-poles is adopted to be used as ABSK signal receiver. The zeros are located on the unit circle; the poles are close to the imaginary axis, so the IIR impacting filter has very high Q value and steep resonance peak, as well as high frequency selectivity. The ideal phase-frequency curve of the impacting filter has its steepest slope near the carrier frequency, so as to achieve differential effects on the ABSK signal phase. The tiny change of the carrier's phase can bring about obvious amplitude changes. For an ABSK signal, if the codes “0” and “1” have the same amplitude, the ABSK signal is a PM signal. When the frequency of the carrier is located between the zeros and poles, *i.e.*, the steepest part of the filter's phase-frequency curve, the ABSK signal can achieve the magnitude impact after filtering. Figure 3 shows the magnification of one segment of the SIF's frequency response, where it can be seen that the SIF meets the requirements of “slope-phase discrimination”.

**Figure 4.**
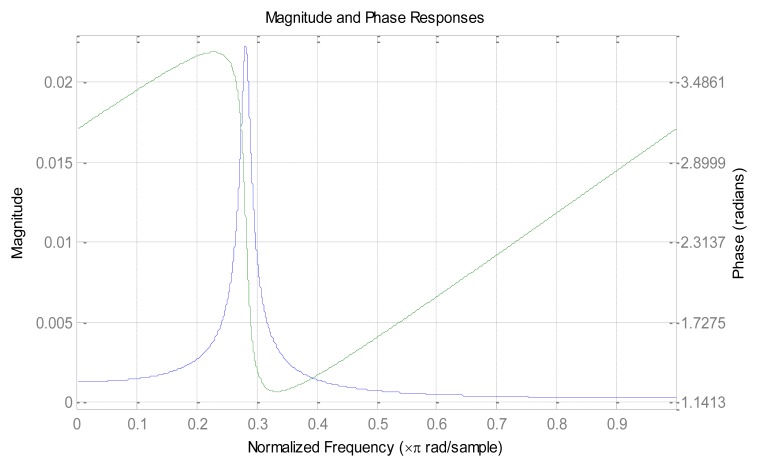
Frequency response of the FIR narrow-band filter.

Based on the above description, there are two key points for the impacting effect: the “slope-phase discrimination” character and the high Q value of resonance circuit. We know that the bandwidth of transmission signal is inversely proportional to the Q value, and thus a larger Q value can lead to narrower transmission bands and better circuit selectivity. Because the FIR narrow-band filter has natural steep transmission bands, we can infer that the ABSK signal's carrier frequency can be placed on the steepest slope of the transmission bands to obtain the “slope-phase discrimination” effect. The frequency response of the FIR narrow-band filter satisfies the condition of “slope-phase discrimination”, as shown in Figure 4.

**Figure 5.**
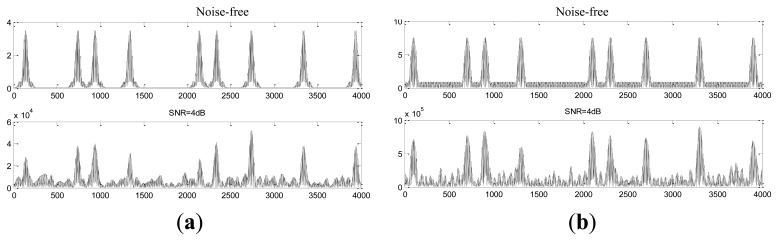
The impacting effect of the FIR narrow-band filter. (**a**) The impacting effect of left transmission band. (**b**) The impacting effect of right transmission band

Figure 5 shows the impacting effect of the FIR narrow-band filter. It can be seen that the code “1” can be impacted after filtering, whether the carrier frequency is located in the left or right transmission bands of the FIR filter.

## 3. FIR Narrow-Band Bandpass Filtering System

**Figure 6.**
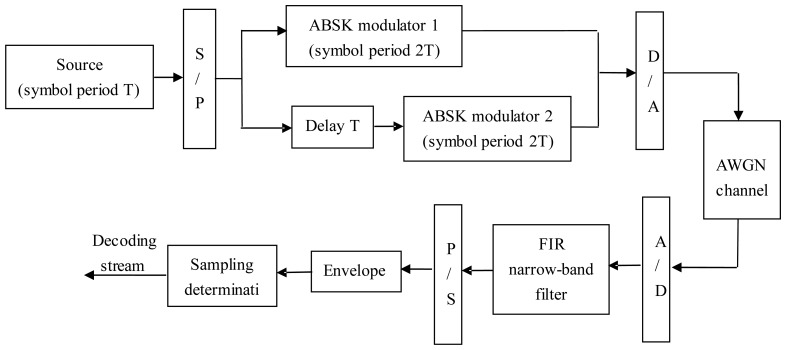
Dual-carrier ABSK communication system.

In order to increase the transmission rate and restrain the inter-symbol and multipath interference, the practical communication system usually use the multi-carrier parallel transmission, such as OFDM, which decomposes the data flow to several sub-data flows, and thus the sub-data flows have much lower transmission rates. A multi-carrier ABSK communication system has been proposed in [10], where the receive filter bank contains at least two SIFs, and each sub-channel has one SIF cascading with at least one notch filter. The dual carrier ABSK communication system used in this paper has two subcarriers. The traditional demodulation of ABSK multi-carrier system is complex; each sub-channel has its own demodulation process, which includes SIF, carrier synchronization, sampling synchronization, bit synchronization, envelope detection, and sample decision respectively. Furthermore, the performance of the complex receiver with IIR structure is unstable. However, the impacting effect of FIR narrow-band filter shown in Figure 5 can be considered to replace the IIR SIF banks, and the dual-carrier ABSK communication system based on the FIR narrow-band filter is proposed as follows:

(1) At the transmission end, the respective data bits of the data sequence (denoted T as the symbol period, 2R bps data rate) was alternately modulated on the carrier frequency *f*c_1_ and *f*c_2_, respectively (denoted 2T as the new symbol period, R bps data rate). Set one sub carrier time-delay T to avoid the both carrier's “1” occurs simultaneously, *i.e.*, make the two “1” to be frequency separation and time-division, in order to ensure the sparsity of the demodulated time signal;
(2) At the receiving end, the FIR narrow-band filter is used as receiver, as shown in Figure 4. The two carrier frequencies *f*c_1_ and *f*c_2_ were placed on the left and right transition bands of the steepest slope. Because of the “slope-phase discrimination” character of FIR sideband, symbol “1” can be impacted, and then the envelope detector outputs a 2R bps rate pulse, and realizes code rate doubled in one passband finally. Figure 6 shows the block diagram of the dual-carrier ABSK communication system with digital narrowband filter as its receiver.

**Figure 7.**
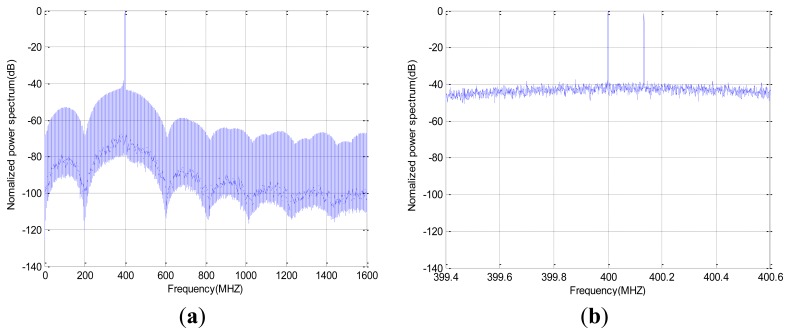
Frequency spectrum of the dual-carriers. (**a**) global figure; (**b**) partial view.

The dual-carrier ABSK signal's frequency spectrum is shown in Figure 7. It can be seen that the dual-carrier system has efficient usage of spectrum.

## 4. Simulation and Numerical Results

In our work, the EBPSK modulated signal is chosen as the transmit signal (set A = B = 1, θ = π) [11–16], which can be described as follows:

(7) $$\begin{array}{ll} f_{0}\left(t\right) & =A\sin\omega_{c}t,0\leq t<T\\ f_{1}\left(t\right) & =\left\{ \begin{array}{l} B\sin\left(\omega_{c}t+\theta \right),0\leq t<\tau ,0\leq \theta \leq \pi \\ A\sin\left(\omega_{c}t\right),\tau \leq t<T \end{array}\right. \end{array}$$

Let us consider the following specifications of the system:

Bit rate R = 1 M bps (symbol period T = 10^−6^ s);
Carrier frequency *f_c_* = 400 MHz;

The bit-rate of each sub-channel EBPSK signal is 0.5M bps, the parameters of the two modulated EBPSK signal are given by: *f*_c1_ = 400 MHz, K_1_:N_1_ = 2:400; *f*_c2_*_i_* = *f*_c1_ + Δ*f*_c_*_i_*, *I* = 1,2,3,4; Δ*f*_c1_ = 0.002HMz, Δ*f*_c2_ = 0.004 MHz, Δ*f*_c3_ = 0.006 MHz, Δ*f*_c4_ = 0.01Hz, the duty K_2_*_i_*:N_2_*_i_* changed with *f*_c2_*_i_*.

**Figure 8.**
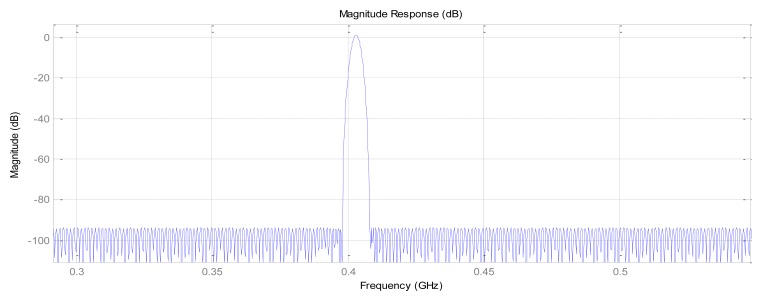
Amplitude-frequency response of the FIR narrow-bandpass filter.

“Slope-phase discrimination” is mainly manifested in the dramatic changes of magnitude brought by the phase changes, so the construction of extremely steep narrow transition bands of the FIR filter is very necessary. The realization of the construction is through comprehensive selection of the pass-band frequency *f*_pass_, stop-band frequency *f*_stop_ and pass stop-band attenuation coefficient *a*_pass_ and *a*_stop_. Here, we present one design example of FIR narrow-band filter to demonstrate the effectiveness of the proposed principle. Set *f*_c1_ = 400 MHz, *f*_c24_ = 400.01 MHz, according to the above principles, Kaiser Window is chosen to realize the FIR filter, set *f*_s_ = 1.2 GHz, transition bandwidth 0.005 MHz, pass stop-band fluctuations 0.01. Specific parameters: *f*_stop1_ = 399.999 MHz, *f*_pass1_ = 400.004 MHz, *f*_pass2_ = 400.006 MHz, *f*_stop2_ = 400.011 MHz, *a*_pass_= −1 dB, *a*_stop1_ = *a*_stop2_ = −100 dB; the frequency response of the FIR narrow-band filter is shown in Figure 8, where it can be seen the steep slope of about 90° fluctuates near the transition bands.

**Figure 9.**
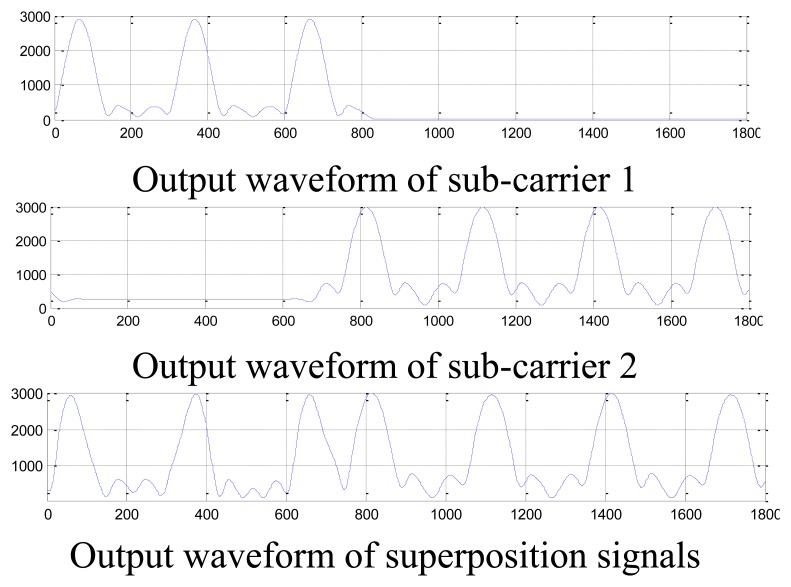
The output waveforms of the FIR narrow-bandpass filter.

Let the two independent signals and their superposition signals pass the designed FIR narrow-bandpass filter, and the filtering results are shown in Figure 9. It can be seen the designed FIR narrow-bandpass filter could impact not only the single-carrier signal, but also the dual-carrier signals.

## 5. Performance

**Figure 10.**
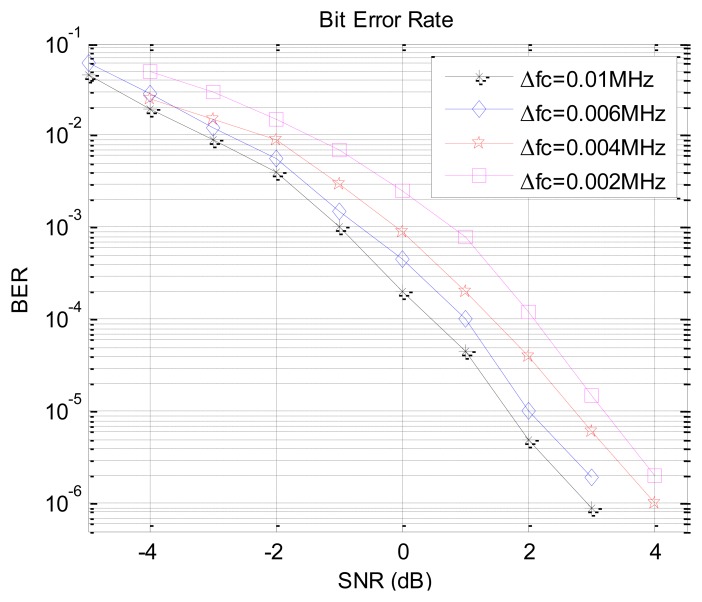
BER performance of the ABSK dual-carrier system.

Without channel coding and bandpass shaping in the additive white Gaussian noise (AWGN) channel, the ADC sampling frequency of receiver is 1.2 GHz, and the proposed FIR narrow-band filter is used to filter the received signal. The BER performance of the EBPSK dual-carrier system is shown in Figure 10.

As shown in Figure 10, with the interval of *f*_c1_ and *f*_c2_*_i_* increasing, the mutual interference between two EBPSK signals becomes much lower and the system demodulation performance becomes much better. While the frequency interval is 0.01 MHz, the SNR (signal to noise ratio) sensitivity is 1.7 dB at the BER of 10^−5^. Even for the other three smaller carrier frequency spacing, the required SNR is no more than 4 dB.

## 6. System Performance Comparison with IIR SIF

**Figure 11.**
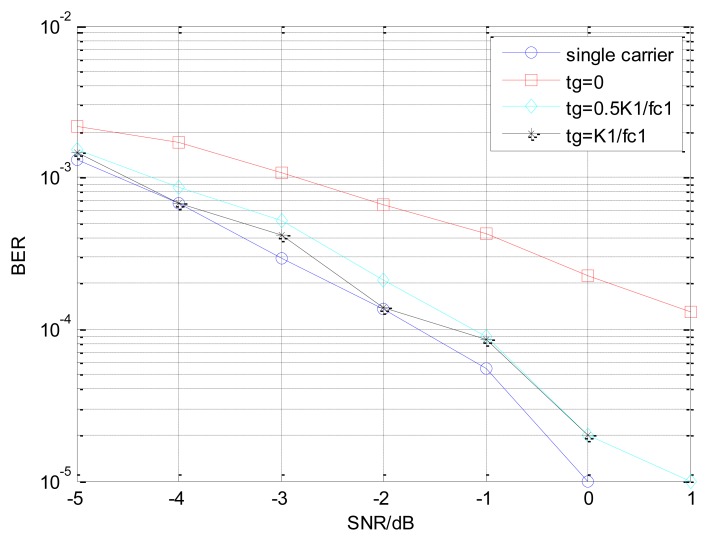
The BER performance of dual-carrier EBPSK system with SIF.

Figure 11 shows the BER performance of the dual-carrier system which adopts the time division method to demodulate the two channels, with the IIR SIF as its receiver. The carrier cycle of sub-carrier is same to the first carrier's, but the second channel has a transition delay t_g_. The system demodulates the two channels of signal respectively, which suppresses the one channel to demodulate another. The frequency interval between the dual-carrier is 0.1 MHz, and the t_g_ of the second channel gets 0, 0.5 K_1_/*f*_c1_, K_1_/*f*_c1_ respectively. As shown in Figure 11, the BER performance becomes better when the time delay is increased. The BER performance of FIR narrow-bandpass filter system is almost the same as the IIR digital filter system, while t_g_ = 0, but the structure of the former is much simpler.

## 7. Conclusions

Simulation results show that the proposed FIR narrowband filter used in the receiver can not only achieve high spectral efficiency, but also give excellent demodulation performance, compared to the previous IIR digital filter bank. The proposed filter also has the following advantages:

(1) Enhanced system stability: the receiver structure has been greatly simplified [17,18];
(2) Compared to the SIF, the proposed filter can quickly transit to the steady state with short transient waveform tail, so the FIR filter can reduce the inter-symbol interference and achieve higher bit rate;
(3) No feedback loop could discard the starting oscillation, which greatly enhance the transmission of small data packet communications efficiency;

We will next design a multi-rate narrowband FIR filter to achieve the performance of an elliptic IIR filter. To further improve the spectral efficiency, we consider using the comb filter at the receiver end to achieve greater capacity for intensive multi-carrier communication.
